# Synchronization of the Normal Human Peripheral Immune System: A Comprehensive Circadian Systems Immunology Analysis

**DOI:** 10.1038/s41598-019-56951-5

**Published:** 2020-01-20

**Authors:** Craig A. Beam, Clive Wasserfall, Alyssa Woodwyk, McKenzie Akers, Heather Rauch, Thomas Blok, Patrice Mason, Duncan Vos, Daniel Perry, Todd Brusko, Mark Peakman, Mark Atkinson

**Affiliations:** 1 Department of Biomedical Sciences, Western Michigan University Homer W. Stryker M.D. School of Medicine, Kalamazoo Michigan, USA; 2 Department of Pathology, Immunology, and Laboratory Medicine, University of Florida Diabetes Institute, Gainesville, FL USA; 3 Center for Clinical Research, Western Michigan University Homer W. Stryker M.D. School of Medicine, Kalamazoo Michigan, USA; 4 Department of Immunobiology, Faculty of Life Sciences & Medicine, King’s College London, 2nd Floor, Borough Wing, Guy’s Hospital, London, SE1 9RT UK

**Keywords:** Immunological techniques, Immunology, Systems analysis, Biomarkers, Translational research

## Abstract

In this study, we sought to fill an important gap in fundamental immunology research by conducting a comprehensive systems immunology analysis of daily variation in the normal human peripheral immune system. Although variation due to circadian rhythmicity was not a significant source of variation in daily B-cell levels or any CD4+ functional subset, it accounted for more than 25% of CD4+ regulatory T-cell variation and over 50% of CD8+ central memory variation. Circadian rhythmicity demonstrated phase alignment within functional phenotypes. In addition, we observed that previously-described mechanistic relationships can also appear in the peripheral system as phase shifting in rhythmic patterns. We identified a set of immune factors which are ubiquitously correlated with other factors and further analysis also identified a tightly-correlated “core” set whose relational structure persisted after analytically removing circadian-related variation. This core set consisted of CD8+ and its subpopulations and the NK population. In sum, the peripheral immune system can be conceptualized as a dynamic, interconnected wave-field repeating its pattern on a daily basis. Our data provide a comprehensive inventory of synchronization and correlation within this wave-field and we encourage use of our data to discover unknown mechanistic relationships which can then be tested in the laboratory.

## Introduction

Immune variation, in both circulating cell frequency and phenotype, have been associated with multiple autoimmune diseases^1^. For example, variations in subsets of immune cells have been correlated to metabolic changes in type 1 diabetes^2,3^ and found predictive of disease progression in multiple sclerosis^4^. Additionally, peripheral blood immune cell variations were demonstrated to be predictive of a post-vaccination response^5^. Over the long-term, variation within an individual appears to be relatively stable compared to variation between-individuals^6^.

Shorter-term variation occurring within the course of a day (i.e., circadian) is now a well-established characteristic of human immunity in circulation^7–15^. The presence of a “master clock”, entraining the organism to external light cues via the suprachiasmic nuclei of the neuroendocrine system, is thought to be a major but not exclusive driver of the circadian behavior seen in certain components of the human immune system. Circadian changes in immune cell function and abundance within the circulatory system are also thought to result from transcriptional and posttranslational feedback loops generated by a set of interplaying clock proteins and time-keeping clock genes^16^. These “peripheral clocks” and their transcription/translation are now believed to function in nearly every human cell^17^. Immune factors such as cytokines can also influence the circadian clock, providing bidirectional flow of circadian information between the neuroendocrine and immune system^18^. Mavroudis^18^ hypothesized that this network of neuroendocrine-immune interactions consist of complex and integrated molecular feedback and feed-forward loops that function in synchrony in order to optimize immune responses.

A form of “Systems Immunology” was recently introduced by Davis, Tato, and Furman^19^ as a way to understand the immune system as a network and thereby to understand immunologic diseases as a perturbation of this network. In this model, the focus is on immune cells and molecules used for communication and how they collectively respond to perturbations induced by environmental stimuli such as vaccines, antibiotic treatment, diet, commensal microbiota and infections. Although systems biology^20^ has been used to better understand the mechanisms of circadian rhythmicity within specific cell populations^21^, to the best of our knowledge there has not yet been published the type of systems immunology analysis as suggested by Davis *et al*. focused on the cell and cytokine as the basic unit in the human circadian immune system.

Therefore, in this study, we sought to fill this important gap in our fundamental understanding of immunology. Beyond this, we also sought to document the interrelationships between immune cell populations and cytokines as they vary in circulation throughout the course of a 24-hour period to provide a roadmap for future investigations into mechanisms underlying immune-mediated and immune-regulated diseases. Finally, we sought to analytically separate observed circadian-based variation from what we hypothesize to be an underlying circulatory immune profile that is not significantly influenced by time-related external or internal factors.

## Results

Six males and four females ranging in age from 22 to 38 years provided informed consent and were enrolled in the study (Supplementary Table 1**)** which was performed in accordance with a Western Michigan University IRB approved protocol (BMH-2016-0853). Subjects reported having no significant medical history (including past diseases/illnesses, surgery, complications, or trauma), and screening physicals were unremarkable. All subjects were afebrile and asymptomatic at the time of enrollment, altogether supporting their designation as controls having a “normal” human immune system. Body Mass Index (BMI) ranged from 23.69 to 34.58 kg/m^2^ with a mean of 28.73 kg/m^2^. Nine subjects completed all 6-blood specimen collections, which were taken at 4-hour intervals starting at 09:00 hours. One female subject withdrew from the study after the first blood collection.

### Circadian variation of the normal human immune system

**Figure 1:**
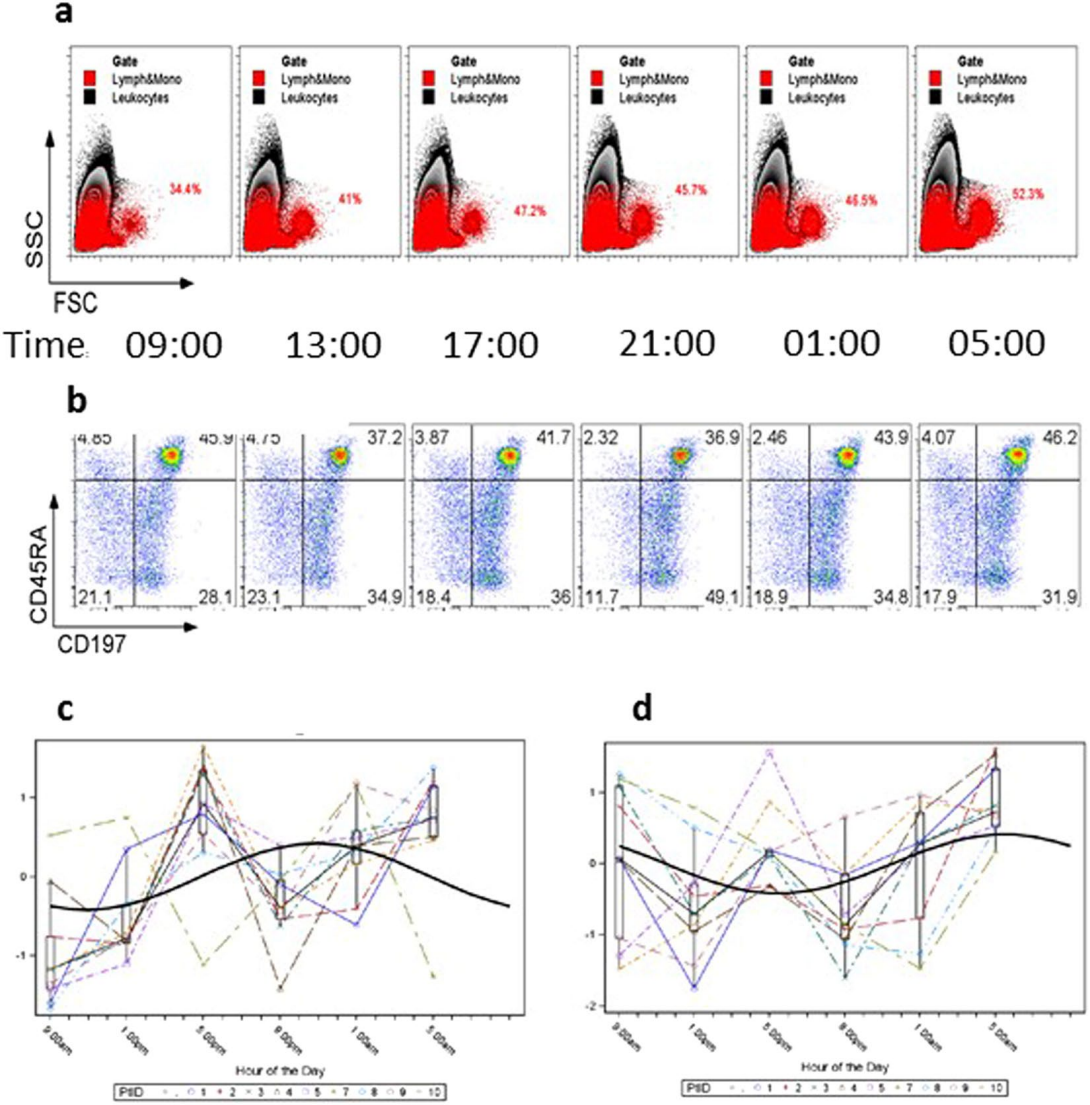
Immune Variation over a 24-Hour Period. Leukocyte variation (panel a) and CD8+ T cell variation (panel b) relative to time (military) are displayed for two study subjects. Panels (c,d) Box plots of leukocytes (**c**) and CD8+ Tcm cell (**d**) variation for each of the 9 study subjects with superimposed cosine curve representing average circadian rhythmicity are presented. Within each box plot the median is presented by a horizontal bar and medians are connected by line segments. In addition, the data from each subject are connected with dashed lines.

Normal circadian (24-hour) variation in immune cell population frequencies and cytokine levels was found to be appreciable within certain individuals as well as, in some cases, for the cohort average. Figure 1 displays flow plots from two individuals: in one subject (panel a), lymphocytes (% leukocytes) varied from 34.4% to 52.3% in the course of the 24-hour period of observation while in another subject (panel b), CD8^+^ central memory T cells (% CD8+, “Tcm”) varied from a minimum of 28.1% to a maximum of 49.0%. Panels c and d of Fig. 1 show the 24-hour variation in these two populations within- and between all subjects. A superimposed smooth curve shows the circadian rhythmicity pattern that was fit to the data (see Methods). It is important to note that while the fitted curve is often near the median of the data, this is not always the case. In addition, these two panels illustrate the appreciable variation that was observed between individuals in their circadian patterns.

Platelets, granulocytes, and lymphocytes were the most variable among the major circulating blood populations, demonstrating daily ranges within individuals (maximum %-minimum %) of 38.60%, 10.75% and 10.75% respectively for the average individual (Table 1). Within the adaptive immune subsets, naïve CD8 T cell frequencies varied by as much as 20.3% within the same individual during the course of a day and average ranges in the CD8^+^ compartment tended to be the largest among the adaptive immune populations. Some populations, such as regulatory CD4^+^ T cells (Tregs) and natural killer T (NKT) cells varied much less over time with the average range for each population being 0.82%. Appreciable cytokine variation was also observed. IL-4 measured from plasma had demonstrable circadian fluctuation: for the individual with the greatest variation, the range was 46.11 pg/mL and the average individual ranged 21.19 pg/mL. In contrast, plasma based IFN-γ apparently had much narrower variation (largest individual range of this cytokine = 8.61 pg/mL with mean individual range = 3.03 pg/mL). Plasma IL-2 was very stable across the 24-hour period with the largest individual range = 2.34 pg/mL and average range = 1.11 pg/mL which is near the lower end of detection. For each soluble analyte measured from both serum and plasma, the range of variation was greater in the latter (Table 1), but the potential significance of this finding as it relates to the use of serum-based vs. plasma-based biomarkers requires further examination.

**Table 1:** Range of daily variation within subjects.

Parameter	Minimum	Maximum	Mean	ICC
WBC (10^3^/μL)	0.90	3.20	1.64	83%
Platelets (10^3^/μL)	18.00	97.00	42.89	91%
Neutrophils (10^3^/μL)	6.00	23.40	11.09	8%
Lymphocytes (10^3^/μL)	4.40	27.60	10.34	63%
Monocytes (10^3^/μL)	1.40	5.70	2.58	76%
Eosinophils (10^3^/μL)	0.50	1.50	1.01	83%
Basophils (10^3^/μL)	0.20	0.40	0.28	68%
Granulocye % of Leukocytes	6.60	18.30	11.94	67%
Lymphocyte % of Leukocytes	6.30	18.40	11.74	67%
B cell % of Lymphocyte	1.57	7.17	3.35	91%
DC % of Lymphocyte	0.10	1.43	0.44	22%
Monocyte % of Lymphocyte	2.30	8.00	5.16	70%
Classical % of Monocytes	5.90	13.20	9.48	48%
Nonclassical % of Monocytes	5.90	13.20	9.49	48%
NK % of Lymphocyte	1.29	16.16	4.43	71%
CD56dim % of NK	1.70	11.60	5.96	67%
CD56bright % of NK	1.64	11.57	5.95	67%
NKT % of Lymphocyte	0.24	2.94	0.91	92%
CD4 Tconv % of Lymphocyte	1.70	17.00	6.98	87%
Naive % of CD4 Tconv	3.40	7.80	5.90	98%
Tem % of CD4 Tconv	1.25	5.00	2.44	97%
Tcm % of CD4 Tconv	1.09	6.00	4.45	98%
Temra % of CD4 Tconv	0.03	5.70	0.93	98%
Treg % of Lymphocyte	0.42	1.54	0.91	49%
CD8 T cell % of Lymphocyte	0.88	7.00	2.46	92%
Naive % of CD8 T cells	7.00	20.30	11.16	86%
Tem % of CD8 T cells	3.48	13.10	8.80	88%
Tcm % of CD8 T cells	1.71	21.00	6.72	89%
Temra % of CD8 Tcells	0.90	19.30	5.66	96%
CD4-CD8- T cell % of Lymph.	0.30	1.45	0.80	81%
CD4+CD8+ T cell % of Lymph.	0.10	2.55	0.65	89%
Unidentified % of Lymphocyte	2.20	11.70	4.32	83%
Serum IFNγ	1.39	7.47	3.37	94%
Serum IL 12	0.95	2.76	1.57	96%
Serum IL 2	0.40	2.82	1.21	97%
Serum IL 4	4.37	28.15	12.75	97%
Serum IL 6	0.58	3.27	1.83	99%
Plasma IFNγ	0.87	8.61	3.36	96%
Plasma IL 12	0.77	4.37	2.19	95%
Plasma IL 2	0.37	2.34	1.24	98%
Plasma IL 4	7.26	46.11	23.54	80%
Plasma IL 6	0.73	5.08	2.20	97%
Serum sIL 2Ra	103.61	326.00	183.39	94%
Serum sIL 4 R	111.00	1566.00	515.78	72%
Serum sIL 6 R	2544.00	17085.00	7597.44	86%
Plasma sIL 2Ra	72.31	306.00	173.95	91%
Plasma sIL 4 R	96.00	1278.00	383.22	84%
Plasma sIL 6 R	4223.00	12112.00	6578.33	84%
cortisol	8.82	25.10	12.97	39%

For each component, data presented are minimum, maximum and mean of the ranges found across all time points from each of the 9 subjects. Units: Cell populations (%), soluble factors (pg/mL). Abbreviations: DC = dendritic cell; NK = natural killer cell; Tconv = Conventional T-cell; Tem = effector memory T-cell; Tcm = central memory T-cell; ICC = intraclass correlation.

Table 1 also reports values of the “ICC” or “Intraclass Correlation Coefficient”, a measure of the percent of total variation that is attributable to differences between subjects rather than variations within the same individual. For example, nearly all (98%) of the daily variation observed in the frequency of naïve CD4+ T-cell population is estimated to come from differences between subjects and not the variation within subject, a component of which might be due to circadian-based rhythmicity. In this case, we regard circadian variation to not be biologically significant. In contrast, more than one-half of the daily variation in regulatory T-cells is due to variation within the subject, a value we consider biologically significant, and some of which might be attributable to circadian rhythmicity. To strengthen the interpretation of our findings, we have therefore decided to only further consider those immune factors in which within subject variability is at least 10% of the total variation of the factor, a level we will denote as “biologically significant”. Since circadian-based variation is no greater than within subject variation, we therefore observe from Table 1 that circadian variation is not a biologically significant source of daily variation in B-cells, NKT-cells, CD8+ T-cells or any of the functional subsets of the CD4+ T-cell populations. However, the daily variation observed in granulocytes, lymphocytes, monocytic lymphocytes, conventional CD4+ T-cells, CD8+ subsets (naïve, central and effector memory), DC, NK (and CD56 dim and bright subsets), regulatory T-cells were biologically significant.

### Normal immune circadian variation is differentially explained by circadian rhythmicity

For almost every immune parameter studied, a cosine curve fitted to the observed circadian data was statistically significant at the p < 0.05 level (Supplementary Table 3). It is noteworthy that the neuroendocrine hormone cortisol, an agent of the master circadian clock^14,22^, had little effect on the significance of the fitted cosinor curves since its inclusion or exclusion in the Cosinor analysis often did not alter interpretation of circadian rhythmicity.

**Figure 2:**
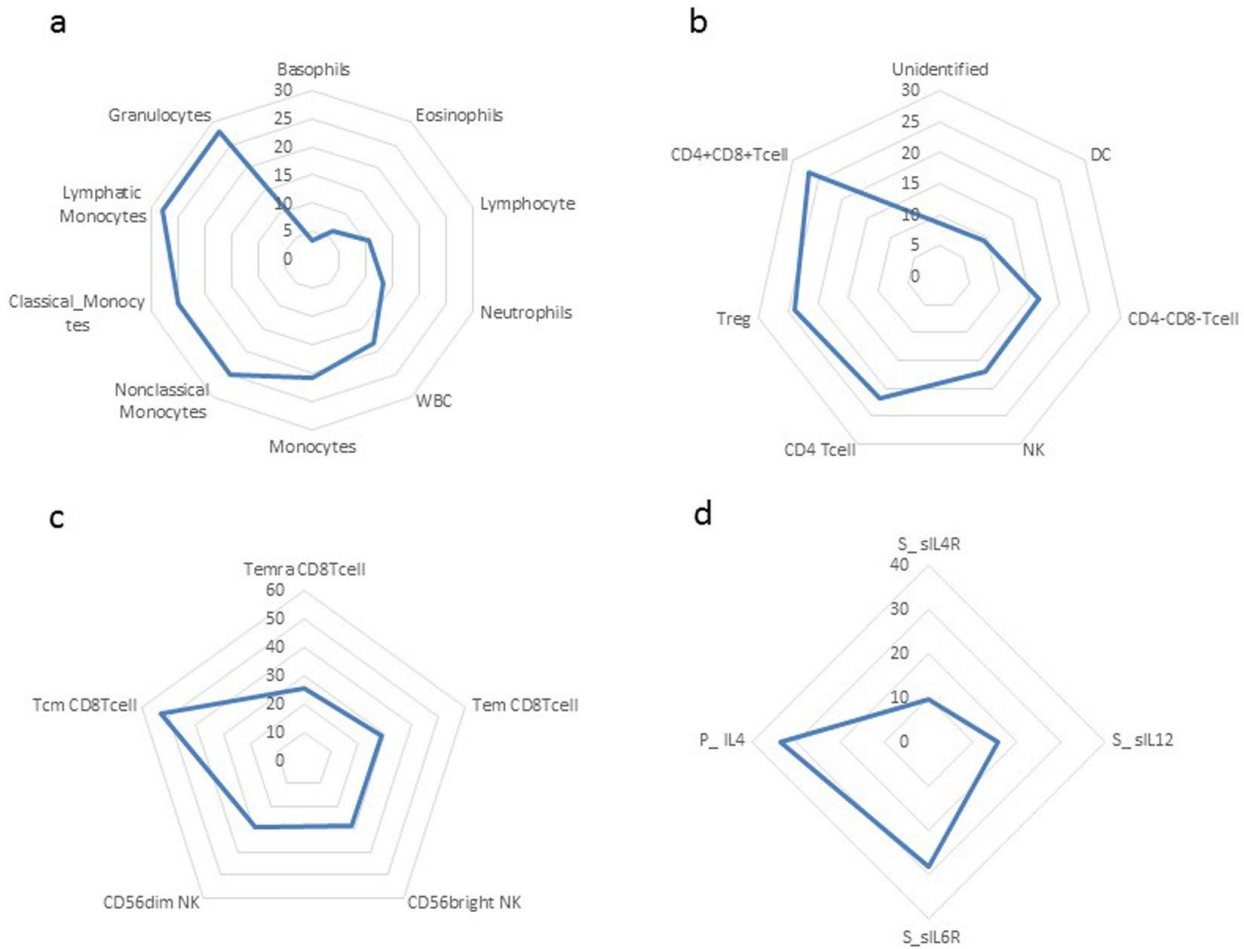
Percent Within-Subject Variation Attributable to Circadian Rhythmicity. Radar plots are used in each panel to show the percent total variation that can be explained by rhythmicity. In those immune factors having biologically significant variation. Each plot consists of a series of concentric rings representing levels of the percentage of daily variation that can be attributed to circadian rhythmicity. Observed values are plotted clockwise in increasing order and connected by a smooth line. Panel (a) Major circulating blood populations; Panel (b) Major innate and adaptive immune populations; Panel (c) Adaptive immune subpopulations; Panel (d) Plasma cytokines (“S” denotes serum; “P” denotes plasma).

For those immune factors having biologically significant daily variation, the percentage of that daily variation estimated to be attributable to circadian rhythmicity are presented in Fig. 2. Daily variability due to circadian rhythmicity was found to be minimal (<10%) for basophils, eosinophils in panel a, DC in panel b and serum levels of soluble IL-4. Otherwise, circadian related variation was significant (≥10%). Of note, it is estimated that approximately one quarter of the daily variation in Treg (panel b), and over 50% of CD8 central memory variation, is attributable to circadian rhythmicity (panel c).

### As a system, daily variation in the normal human peripheral immune system can be conceptualized as a wave-field

**Figure 3:**
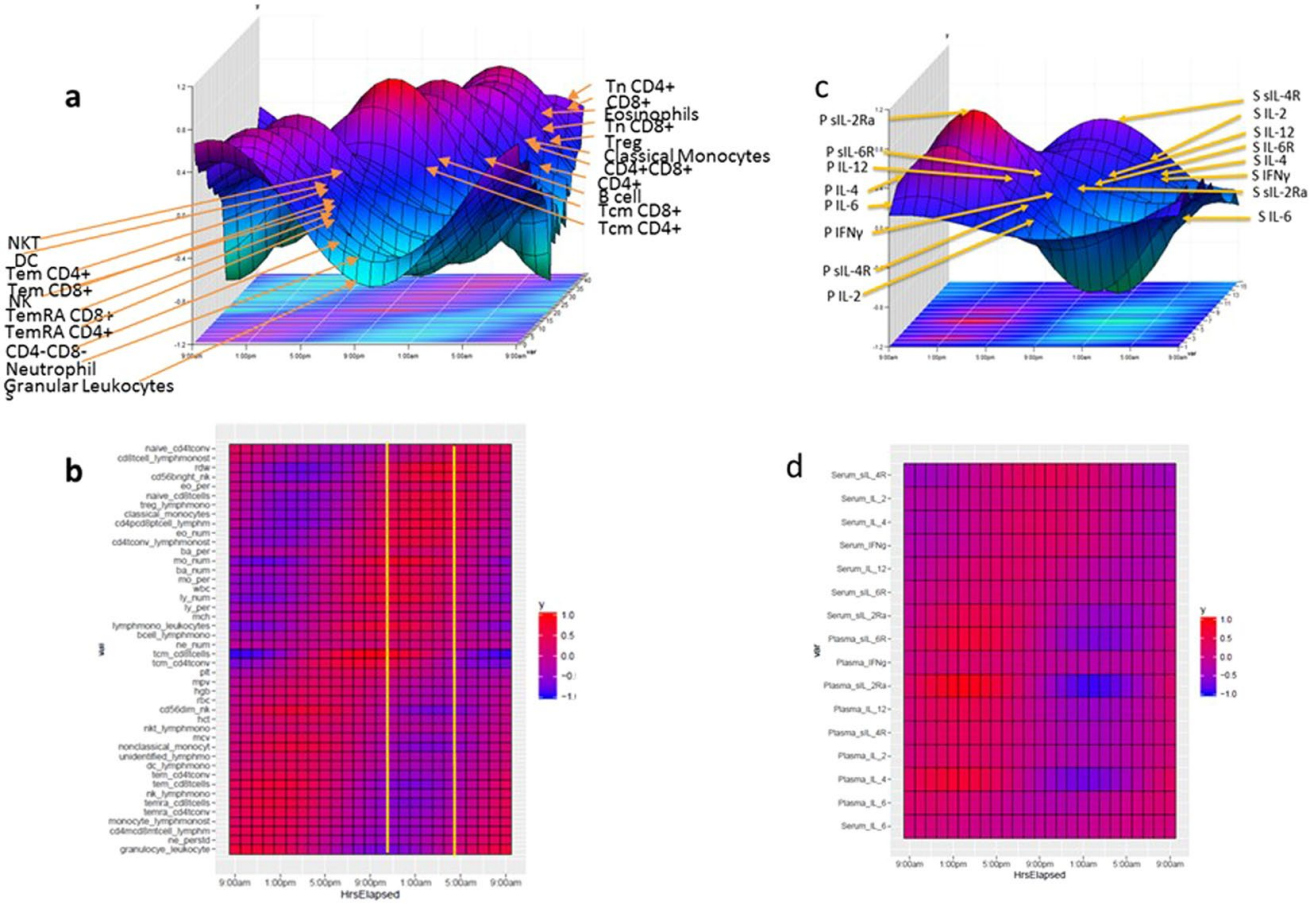
Systems Immunology Characterization of the Normal Human Immune System. Panels (a,b) −24 hour rhythmicity of key cellular (**a**) and cytokine (**c**) immune components arranged in order of the time of their peak. Vertical axis presents standardized cell frequencies (**a**) and standardized cytokine levels (**d**). Horizontal axis represents time of collection beginning at 9 am. A heatmap is displayed on the lower plane of the graph and reproduced for greater clarity in panels b and d. Heatmap color gradations denote level of standardized values of cell frequency (**b**) or cytokine level (**d**). The range of time in which peak regulatory CD4+ T-cells occur is indicated by two yellow lines on panel b. In panel c, “P” denotes plasma and “S” denotes serum derived measurements.

When taken together (Fig. 3), the rhythmicity patterns appear to align in a sequentially-ordered series of distinct peak and trough levels suggestive of a temporally unfolding process possibly arising from a sequential series of timed mechanistic interactions. As suggested by Mazzoccoli^13^, these mechanistic interactions may have been evolutionarily arranged to maximize immune system homeostasis and function. Some subsets of CD4 and CD8 T cell populations (Fig. 3 panel a) peak near each other: specifically, CD45RA^-^CD197^-^ T effector memory (Tem), CD45RA^+^CD197^−^ Temra and CD45RA^−^CD197^+^ Tcm subsets. A similar pairing in time was also observed in HLA-DR^+^CD14^−^CD16^−^ DC and CD3^+^CD56^+^ NKT cell populations.

If we separate the 24 hour observation period into “day” (times of sunrise-sunset on the day of our study: approximately 6 am–6 pm) and “night” (6 pm–6 am) segments, we observe that innate and effector memory cells generally peak during the day while Tregs, naïve memory, Tcm cells and B cells generally peak during the night which is consistent with previous research^13^. High levels of many populations extend over several hours (Fig. 3 panel c). During the period of high and peak levels of Tregs (indicated by yellow vertical lines on Fig. 3 panel c), innate immune cells (NK and DC) and Tem populations (CD4^+^ and CD8^+^) had low frequency and reached their nadir. On the other hand, naïve members of both the CD4 and the CD8 T cell populations had high levels and experienced their peak near Treg peak. Cytokine variation also exhibited circadian rhythmicity that can be described by cosine curves (Fig. 3 panels b and d). It is interesting to note that while low levels of IL-4 corresponded with the peak Treg period (i.e., night), its peak occurred soon after this period.

### Circadian rhythmicity demonstrates phase alignment within functional phenotypes

**Figure 4:**
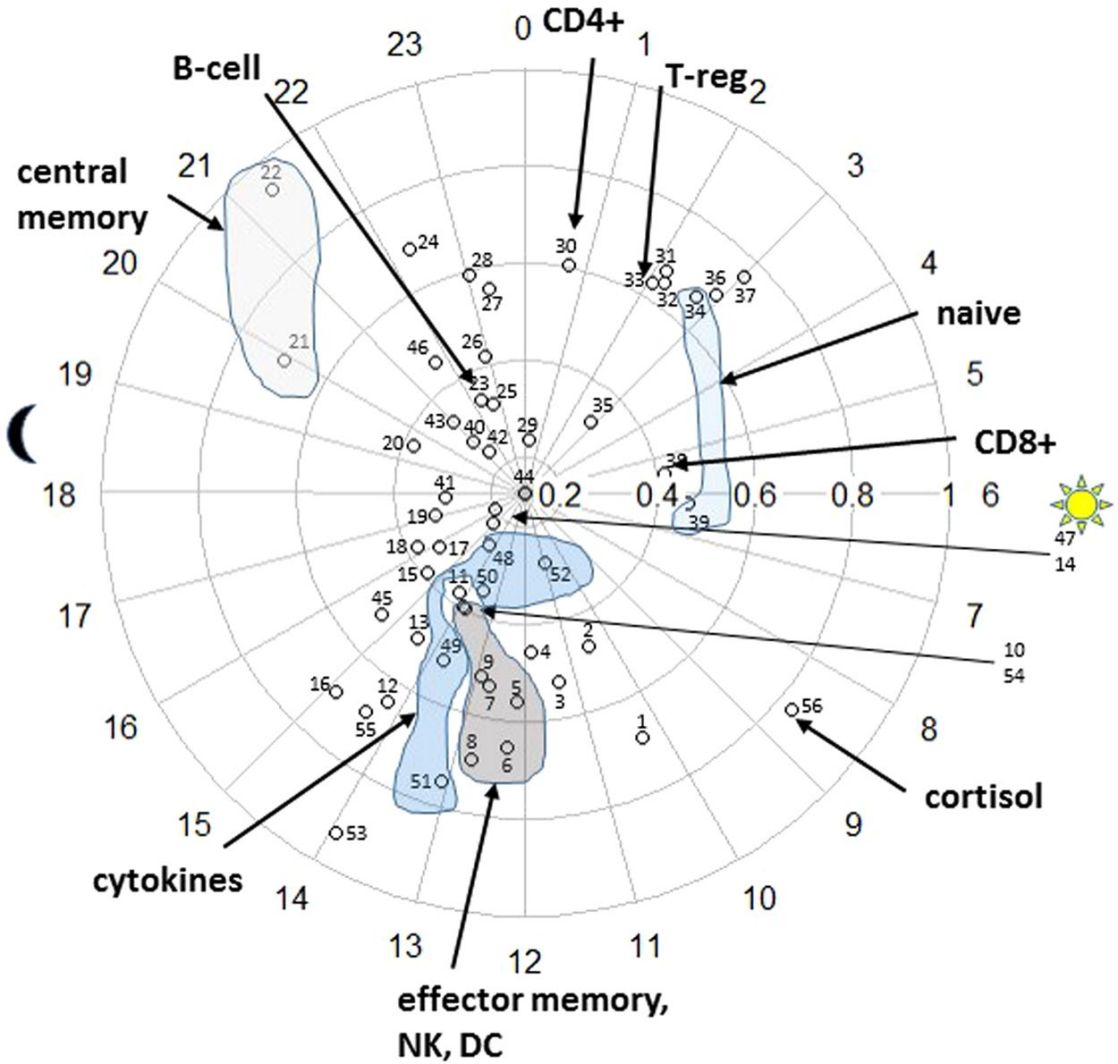
Phase Alignment among Functional Phenotypes. Each numbered circle represents an immune or hemodynamic factor measured in this study (key in Table 4 of Supplemental Material). Placement of the circle on a 24-hour “clock” indicates acrophase (time of peak). Amplitude, or height of peak (expressed in standard deviation units), is indicated by distance from clock center. Time of sunrise and sunset occurring during the study are indicated by sun and moon symbols. Some data points have been visually grouped by functional phenotype (shaded regions) and are identified as follows: “Central Memory” (data point 21 = CD4, 22 = CD8); “Naïve” (34 = CD8, 39 = CD4); “Effector Memory, NK,DC” (5 = TEMRA CD4, 6 = TEMRA CD8, 7 = NK, 8 = TEM CD8, 9 = TEM CD4, 10 = DC); “Cytokines” (48 = IFNγ, 49 = IL-12, 50 = IL-2, 51 = IL-4, 52 = IL-6).

As described in Methods, circadian-related variation can be completely characterized by three parameters one of which, “acrophase”, denotes the time at which the highest level or “peak” occurs. Figure 4 displays the acrophases of the immune factors arranged on a 24-hour clock. After grouping the immune factors by functional phenotypes, we observe that acrophases of immune factors of similar function occur near one another. Thus, we observe that phase alignment of circadian rhythmicity occurs within functional phenotype.

### Sequential phase alignment and significant correlation identify two major subsystems suggesting new mechanistic relationships

We next examined correlations in order to identify possible new mechanistic relationships among components of the normal human immune system. In addition, we also consider statistically significant correlations signifying a coefficient of determination (i.e. R^2^) of at least 10% to be biologically significant since then at least 10% of the variation in one immune factor is attributable to variation in the other factor.

To aid in discovery, we ordered the components with respect to the appearance of their acrophase (time of peak level), as illustrated in Fig. 3. Results of the analysis of correlations (Supplementary Table 2) show there to be significant positive correlations appearing in many pairs of components whose peaks occur one after the other, termed here “Sequential Phase Alignment (SPA)”. Significant SPA correlation is seen almost exclusively within the adaptive immune compartment. However the notable exception is the innate immune NK population. Importantly, all members of the “SPA” subsystem are significantly and positively correlated. Supplementary Table 2 further emphasizes that the normal human immune system can generally be divided into “day-peaking” and “night-peaking” subsystems and that components within a “diurnal subsystem” tend to exhibit positively correlated SPA while components appearing in different diurnal subsystems appear to be mostly negatively correlated. For example, we observe from Supplementary Table 2 that plasma IL-12 peaks during the day, it is significantly and negatively correlated with the night-peaking components Treg, CD4^+^CD8^+^ T cell and CD56^bright^ NK cell populations.

Perhaps the most important observation to be made from Supplementary Table 2 is the loss of SPA correlation occurring at the times of environmental transitions between light and dark periods. Specifically, although their peaks occur sequentially in time during the transition from day to night, DC and IFNγ have negligible correlation (r = −0.004). Similarly, during the transition from night to day, the correlation between eosinophils and NK bright populations which reach their peaks sequentially, is also negligible (r = 0 0.006). This is in contrast to the fact that most pairs of immune components that peak near each other are statistically significantly different from zero and positive.

### There are ubiquitous correlates within the circadian variation of the normal human peripheral immune system

Several of the twenty-seven immune factors studied were found to be significantly (both biologically and statistically) correlated with the majority (at least 50%) of the other factors as they co-varied throughout the day (Supplementary Table 2). These ubiquitous correlates were: NK, NK CD56^dim^, CD4, CD4 TEM, CD4 TEMRA, CD8 TEMRA and CD4-CD8- populations. Regulatory T-cells were found to be significantly correlated with ten (37%) other immune factors.

### The negative treg/nk immune relationship is reflected by phase-shifting in the periphery

**Figure 5:**
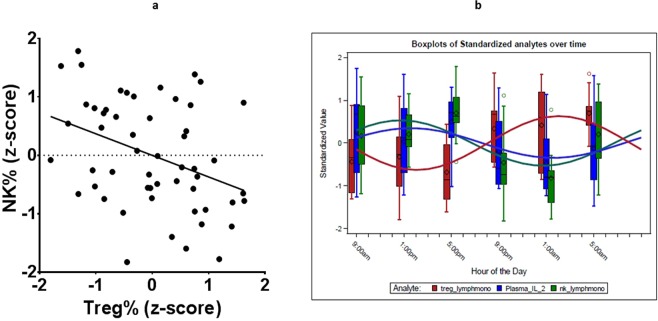
Apparent vs. Circadian Correlation. Panel (a) Scatterplot of standardized NK percent (vertical axis) vs. Regulatory T-cell pecent (horizontal axis). All timepoints from all subjects are presented. A regression line is superimposed. Panel (b) The same data as in panel a are presented in summary form via box plots at each time of data collection. Superimposed are circadian rhythmicity curves fitted to the data. (green = NK; red = Treg;blue = plasma IL-2).

Previous studies have reported a negative relationship between NK and Treg population frequencies^23–32^. Panel a of Fig. 5 shows standardized fequencies of these two immune cell subsets from our data and includes all time points of data collection and all subjects. A fitted regression line substantiates the existence of a negative relationship as does a repeated-measures adjusted correlation coefficient (r = −0.309, p = 0.0241). However, Fig. 5 panel b shows the same data now being displayed across the 24-hour period of the study with fitted cosine curves which cross in opposing cycles. Hence, the negative correlation of Treg and NK cells is seen to possibly arise simply from the alternating circadian rhythmicity of each of these immune components: As Treg levels (red) fall during the morning (approximately 9 am to 1 pm) NK levels rise (green). Throughout the afternoon and evening (approximately 1 pm to 1 am) Treg levels rise while NK levels fall. The decline of Treg in the early morning hours is accompanied by an increase in NK levels and restarts the next cycle. We therefore observe that cicadian rhythmicity could explain the previously reported negative correlation between NK and Treg without requiring any form of cell interaction. That is, this biological phenomenon could occur solely via the central coordination provided by the master clock and/or via coordinated perhiperal clocks, with cells traficking in and out of the peripheral circulation. Of course, if we keep in mind the joint circadian ryhtmicity suggested by Fig. 3, it is also possible that there exists a series of intermediate mechanistic events that occur rhythmically and form a chain between the opposing cycles of NK and Treg cells. One plausible intermediary is competition for the cytokine IL-2^33^, which was found to track closely with NK cells but to have an opposing cycle with T-reg. Competition for IL-2 has been postulated to explain the negative NK-Treg relationship^24,29,33^. It is also thought that Tregs should have the advantage as they constitutively express CD25 forming the high affinity trimeric IL-2 receptor^34,35^ versus NK cells where CD25 expression must be induced upon activation. These suggestions are substantiated by our data and provide an example of a possible mechanistic linkage between the rhythmic patterns of two immune populations.

### Circadian-independent correlation of the peripheral immune system

**Figure 6:**
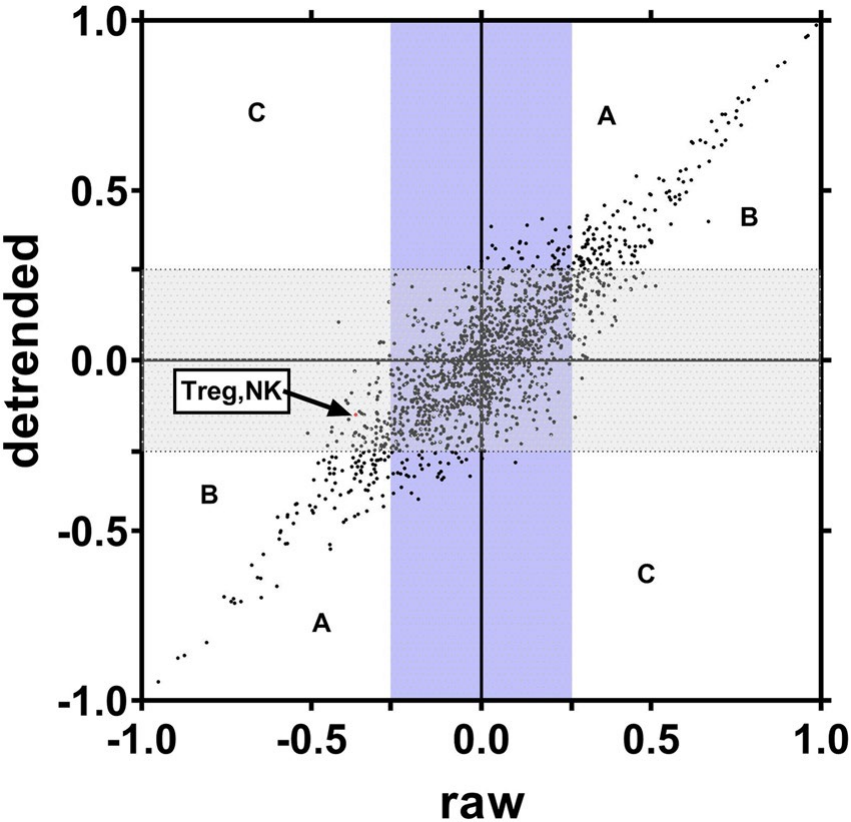
Circadian vs. Detrended Correlations. Horizontal (“x”)-axis represents correlations between each possible pair of immune parameters. Y-axis represents these correlations after removal of circadian variation of the each immune parameter in the pair (“detrended”). Superimposed shaded areas denote correlations which would not be declared as significantly different after adjusting FDR = 10% in this study. Region “A” represents correlations that were significant in both the circadian and detrended data. Region “B “represents those correlations not significant in the detrended data but that were significant in the circadian data. Region “C” represents those correlations significant in the detrended but not the circadian data.

Examination of Figs. 1 and 2 also suggest that not all daily variation is due to circadian rhythmicity. We therefore also examined the correlation among components of the circulating immune system that remained after we statistically removed circadian variation from each component (“detrend”) (see Methods). Figure 6 plots correlations found in the detrended data versus the correlation found in the “raw” (not detrended) data. Superimposed shaded areas denote correlations which would not be declared as significant after adjusting for a False Discovery Rate (FDR) of 10%. Region “A” represents correlations that were significant in both the raw and detrended data. Region “B “represents those correlations not significant in the detrended data but that were significant in the raw data. Region “C” represents those correlations significant in the detrended but not the raw data. As indicated on Fig. 6, the correlation between Tregs and NK cells was significant in the raw (r = −0.370) but not the detrended data (r = −0.161) suggesting that the correlation seen between these two populations in the periphery is largely circadian induced or circadian-amplified. A similar observation can be made for other lymphocytes. For example, the NK CD56^bright^ population was significantly correlated with or CD8 TEMRA (r = −0.349) and classical monocytes populations (r = 0.410) in the presence of circadian variation, but not significantly correlated after this source of variation was removed (r = −0.192 and r = 0.199, respectively).

**Figure 7:**
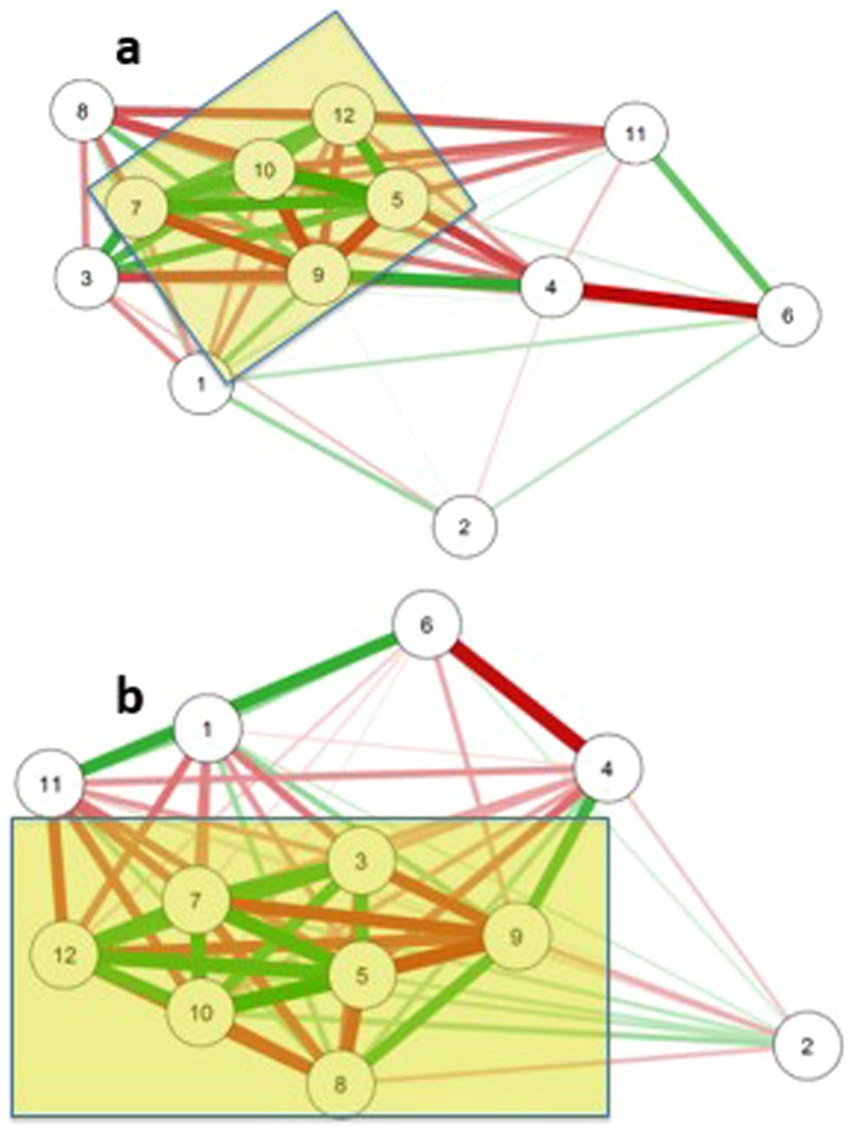
Correlational Clustering of Cell Populations. Thickness of line indicates strength of correlations, green denotes positive and red denotes negative correlation. Proximity indicates clustering. Panel a: circadian correlational structure; Panel b: basal correlational structure. Key: 1-B cell;2-DC;3-NK;4-CD4+ Naïve;5-CD4 TEM;6-CD4 TCM;7-CD4 TEMRA;8-Treg;9-CD8 Naïve;10-CD8 TEM;11-CD8 TCM; 12-CD8.

Figure 7 displays the correlational clustering of adaptive and innate cell populations from the raw (panel a) and detrended (panel b) data. In both cases a “core” cluster is seen (rectangular shaded regions) which is similar between raw and detrended conditions. The “core” is defined by a set of immune cell data whose fluctuations were strongly associated with one another. This common cluster is primarily comprised of effector memory subsets of both the CD4 and CD8 populations, as well as CD8, CD8 Naïve and NK populations and persists in both the raw and detrended data.

## Discussion

Hood^20^ and Davis^19^ first introduced the concepts of systems biology and systems immunology, respectively, as methods to describe the synchronous actions and interactions of a collection of biological units and their methods of communication. Although circadian variations in circulating immune populations have been well documented^7–15^, we believe our study is the first to take the systems approach introduced by Davis and considering these cell populations, soluble factors and their mechansims of communication as the central components of an integrated whole. Our work therefore meets a need to address the synchrony of the human immune system since previously published data have largely been comprised of “reductionist” studies focused on the mechanistic interactions of isolated subsets resulting in a disjointed understanding of human immunity^18^.

A unique aspect of our application of systems immunology is the dimension of time and the subsequent dynamical characterization of the network. This aspect led us to develop a novel conceptualization of the circulating immune system as an integrated wave-field exhibiting an apparently sequentially unfolding structure throughout a 24-hour period. In keeping with previous research^13^, we also observed the general process to favor high levels of defense components (e.g., Tem, NK cells and cytokines) during the day when human activity is typically high and exposure to infectious agents is most likely versus favoring low levels of defense components and high levels of regulatory populations (e.g., Tregs) during the human resting phase in the evening.

The apparently sequential nature of the immune system was discovered by ordering our wave-field model with respect to the time of peak cell population or cytokine level. Sequential pairing along this wave-field then identified significant correlations among most adaptive immune components and established the possibility of sequential mechanistic interactions within this subsystem. This ordering, if replicated, could provide new insights into downstream effects within the system and perhaps new mechanistic models based on time. It seems to us very reasonable to expect that mechanistic relationships among cell populations, even if they only occur in lymph nodes and primary lymphatic tissues, require a period of time for change in input to result in change in output that is subsequently observed in circulation via circadian-timed trafficking^10^.

Our examination of the relationship between Tregs and NK cells is a prime example of the importance of taking a time-related systems immunology approach to human immunology. Previous research, based on static or (cross-sectional) *ex vivo* studies have established mechanistic explanations supporting the negative correlation between Treg and NK cell frequencies. When we examined their relationship across time, we observed they did have opposing rhythmic cycles across a 24-hour period, a phenomenon that can be parsimoniously described as “maximal” phase-shifting Furthermore, when we superimposed the circadian rhythmic pattern of IL-2, we observed that it was in phase with NK cells and maximally phase-shifted to oppose the Treg pattern. We interpret this observation to be a substantiation of the “IL-2 competition” hypothesis for the negative Treg/NK relationship but now using a circadian experimental model of peripheral immunity. We propose use of this model to discover potential relationships for experimental validation.

Although others have previously described daily vs. night acrophase differences among immune populations (including a “Time Table” prepared by Mazzoccoli^13^), our work advances this knowledge base by not only showing that immune factors within each “diurnal subsystem” are positively correlated, but also that they are negatively correlated between the subsystems. Further research is needed to better delineate the role intervening mechanisms might play in creating the correlated “dance” of the circadian immune system as depicted in Fig. 3.

Another contribution of our study is what we believe to be the first description of circulatory immune activity that is independent of the variation and correlation induced by circadian rhythmicity. We found that the circadian-independent correlational structure is sometimes similar to, but at other times different from, the correlational structure of the circadian-influenced data. A very important difference occurs with the correlation between Treg and NK cells. As discussed previously, there is a statistically significant (after FDR adjustment) negative correlation (r = −0.370) between these two that is explainable by their joint circadian rhythmicity. However, after removing their circadian-rhythmicity related variation, their correlation reduced more than 50% to the level of no longer being statistically significantly different from zero (r = −0.161). In the case of Tregs and NK cells, this suggests the extreme (but parsimonious) explanation that there is no intrinsic mechanistic relationship between the two but their negative correlation in the circulating milieu has been genetically engineered through the neuroendocrine system to optimize and balance immune readiness and replenishment. Alternatively, it might also be the case that “circadian-imprinting” has *amplified* a pre-existing yet low-level mechanistic relationship between Treg and NK cells as expressed in circulation. Other pairs of parameters demonstrating possible amplification include relationships between the NK CD56^bright^ population and CD8+ TEMRA and classical monocyte populations.

In a smaller number of pairs of immune components, correlation in the absence of circadian-related variation was significant while circadian-related correlation was not. We refer to this as “*masking*”. As a case in point, after adjusting out circadian-related variation, NK cells and DCs were found to have a significant negative correlation (r = −0.408) in the absence of circadian variation but not a significant correlation after the addition of rhythmic circadian variation (r = −0.190). This finding raises the question whether there might be an evolutionary advantage to the dampening of this relationship in healthy human immune systems. There is some evidence showing that NK cells can lyse immature DC cells as well as exert an immunoregulatory function through cytokine production^36^. However, examination of circulating DC and NK cells’ rhythmic patterns show they track together, peaking in the early afternoon and troughing after midnight. Thus we propose that the phenomenon we have observed could be explained as a dampening of a significant circadian-independent relationship through an imposed circadian rhythmicity intended to maximize the immune potency of the immune system during the active phase of the day.

Our study has several potential limitations to be kept in mind: (1) in focusing on circulating blood we have limited our study to less than 2% of the entire immune system. Yet, as stated in the introduction, variations within peripheral blood cellular components correlate with autoimmune disease activity and therapeutic outcomes. Thus, we contend it is an important and appropriate starting point for systems immunology of circadian variations in the healthy human. (2) Our Cosinor analysis assumes a single-phase cosine-like waveform of the data, which is not always the case. Due to its simplicity, this model might provide interpretations which are not conservative. In addition, the detrended data is an analytic derivative and needs validation. (3) We selected our sample size to match conventional study designs in human circadian research using peripheral blood; the resulting limited number of subjects may have reduced precision in our model fitting and estimates of correlation. Hence, future research with statistically-determined sample size should be undertaken. (4) We did not rigorously control for environmental influences, (e.g. ambient lighting) and a clearer picture could perhaps be obtained with stricter controls in place. An adjustment for melatonin level differences (reflecting differences in individual sleep cycles) might also have benefitted our interpretations. Yet we did control for some inter-individual differences through standardization of data on a per-subject basis, and we did attempt to control for sleep-cycle differences using cortisol, which we found did not change our interpretation in most cases. However, the fact that our clinical protocol did not control for environmental factors (e.g. diet, personal sleep patterns, etc.) other than having ambient lighting reduced at 11 pm, was actually intentional. These decisions were made in order to ensure that findings from our study would be relevant to routine blood sampling conducted in clinical settings. Importantly, when compared to studies with rigorous control over these factors, our estimates are strikingly similar. For example, Ackerman^37^ conducted a study in which subjects were selected on the basis of a number of validated sleep questionnaires, excluded if they smoked, required to maintain a strict regular sleep-wake cycle one week prior to the study (compliance was electronically monitored), and were studied in sleep laboratories under strictly-controlled environmental conditions. Yet, their estimate of the time for peak B cell (% of lymphocytes) was 22.18 hours (military) which is extremely close to that found in our study (|−5.8263|*(24/2π) = 22.26 hours). Since other similarities occurred, we conclude that our protocol gives reasonably accurate estimates reflective of the immune system operating in normal conditions. (5) Our immune panel did not capture FoxP3 limiting the specificity of our measurement of Treg frequency. Moreover, additional measurements could give better insight into the main actors of the synchronization we have described (e.g., chemokines). (6) Limitations in our analytic methodologies are discussed in Methods. (7) Finally, since correlation does not prove causation, the anti-phase relationship observed between variables (e.g., Treg-NK) is not a casual explanation.

In summary, our application of a comprehensive systems immunology approach to daily variations observed in the circulating blood of the normal human has led us to propose a novel conceptualization of the immune system as a wave-field, that is, a collection of rhythmically oscillating components that are correlated and likely interacting across time. Further research based on this conceptualization is needed to test its reproducibility, validity and utility and to more deeply investigate time-sequencing beyond simple pairs of immune components At this point in time, we wish to highlight that our description of circadian correlation in the periphery can now serve to identify new mechanistic relationships for further investigations in lymphatic tissue.

## Methods

### Study subjects

Blood specimens were obtained from 10 healthy volunteers during a 24 h inpatient stay at a Phase 1 clinic in Kalamazoo, MI. Blood was drawn every 4 hours over a 24 h period for a total of 6 specimens per subject. The blood specimens were analyzed by the University of Florida Diabetes Institute (Gainesville, FL). Laboratory and clinical data was compiled and analyzed by the Western Michigan Homer Stryker School of Medicine, Division of Epidemiology and Biostatistics. All studies were approved by the Institutional Review Boards at WMED and UF and all methods were performed in accordance with relevant guidelines and regulations.

### Study protocol

After providing informed consent, subjects underwent a history and physical 6 days prior to the study. The main inclusion criteria were males or females between the ages of 18 and 40 years (inclusive) free of clinically significant chronic or acute diseases. Subjects were instructed to refrain from alcohol and maintain a regular sleep schedule for the remainder of the week prior to study participation. Subjects were excluded if they did shift work that required changes in sleep patterns or had traveled between time zones within 1 week prior to study start. Medications, other than acetaminophen, were excluded for 14 days prior to the study.

Subjects were admitted to the clinic at between 7:00 and 8:00 am on the day of the study. They were assessed for acute illness, females underwent a urine pregnancy test, and eligibility was reconfirmed for all subjects. The subjects were allowed an unrestricted diet during the study and no restrictions on caloric intake were made, and caffeinated beverages were permitted. Lunch and dinner were provided along with breakfast on the morning of discharge. Lights out was at 11 pm, limiting light exposure during the evening. Subjects were allowed to sleep per their usual schedule.

Blood samples were obtained via venipuncture at (±30 minutes) 9 am, 1 pm, 5 pm, and 9 pm on Day 1 and 1 am and 5 am on Day 2. Approximately 5.5 mL of blood was drawn at each time point. (2 mL K2 EDTA (lavendar top) tube and 3.5 mL SST (yellow top) tube). Subjects were discharged after the final blood draw on Day 2. Blood samples were shipped ambient to the University of Florida on the same day.

### Whole blood and human immunity factors

Components of whole blood were analyzed with standard laboratory methods. In addition, our analysis considered humoral and adaptive components of the immune system as well as cytokines currently thought to be important to human immunity (enumerated in Table 1). Methods for the measurement of these components are described below.

### Flow cytometry

Direct immunofluorescence surface staining of whole blood was performed with a panel of 11 markers designed to provide a broad profile of immune cell subsets. The panel is a simplified emulation of those described by Maeker *et al*.^38^. Antibodies against the following antigens were used, listed with their corresponding clones: CD3 (SK7) and CD45RA (HI100) from BD biosciences (San Jose, CA), HLA-DR (LN243), CD4 (RPA-T4), CD8 (SK1), CD14 (M5E2), CD16 (3G8), CD25 (BC96), CD56 (HCD56), CD127 (A019D5), CD197 (G043H7) from Biolegend (San Diego, CA). With this panel, it was possible to either directly quantify, or indirectly impute, the following cell subsets: granulocyte, dendritic cell (DC), monocyte, B cell, several T cell subsets (including natural killer (NK), naïve, conventional, memory, and effector), and regulatory CD4+ T cells (Treg). Antibodies were added to whole blood (200 ul/stain), which was then incubated for 30 min at room temperature and protected from light. Afterwards, 2 mL of Fix/lyse 1× (eBioscience, USA) was added and incubated at room temperature for 5 min. Successive washing/centrifugation (5 min, 450 g) steps were performed until hemolysis color completely faded. The pellet was resuspended in 1 mL of staining buffer, briefly vortexed and stored at 4 °C. Samples were acquired within 24 hours on a BD Fortessa cytometer, and data were analyzed by FlowJo™ (Ashland, OR).

### Cytokines

Plasma cytokines were measured using Luminex bead technology. Specifically the Human Soluble Cytokine Receptor Panel- Immunology Multiplex Assay (sgp130, sIL-2Ra, sIL-4R, sIL-6R) and the Human High Sensitivity T Cell Panel (IL-2, IL-4, IL-6, IFNγ, IL-12(p70)) (Merck) Manufacturers protocol was followed.

### Statistical methods

Preparatory to analysis (data not shown), examination of ICC (“intraclass correlation coefficient”, described below) values showed that percentage-based immune parameters tended to have higher ICC than count data indicating that percentage data has removed some of the within-subject circadian variation. This is a reasonable conclusion since some of the variation of the raw count data reflects between-subject variations in population numbers while the percent data factors that out, reporting results essentially as a count per standardized population count (i.e. population count = 100) to yield a percent. As discussed below, we have established a rule for determining whether within-subject variation is “biologically significant” based on the ICC and therefore concluded that the use of percentage data would be a more conservative approach to selection of immune parameters for further study. We therefore restricted our primary circadian analysis to percentage data for the immune populations. A secondary, descriptive analysis of counts for the major blood populations is provided in the Supplementary Fig. 3.

Standardized versions of the variables were analyzed in order to increase the precision of correlation estimates. The measurements of each variable (cell populations and cytokines) were transformed into “z-scores“ by subtracting the mean of the variable and dividing by the standard deviation of the variable. These values can then be intrepreted as the number of standard deviations from the mean and translated back to the original, untransformed values, using the means and standardard deviations of the variable.

Pearson correlations of the standarized data were computed using the CORR procedure in SAS (v9.4, Cary, NC). Control of the overall “False Discovery Rate (FDR)” (the proportion of false postives among the total number of positive findings) was achieved using the “FDRtool” package in R (R Core Team (2017). R: A language and environment for statistical computing. R Foundation for Statistical Computing, Vienna, Austria. https://www.R-project.org/). The FDR method^39^ is an alternative to the Bonferroni approach for controlling the rate of False Positive findings and is more powerful in settings in which many hypotheses are tested simultaneously. In this study, we set the FDR to not exceed 10%. In addition, we also consider statistically significant correlations signifying a coefficient of determination (i.e. R^2^) of at least 10% to be biologically significant since then at least 10% of the variation in one immune factor is attributable to variation in the other factor.

Circadian rhythmicity patterns were estimated using “COSINOR” analysis^40^ implemented in SAS as a linear model. These data were displayed using wireframe plots in R. Satistical significance of the circadian pattern was established by testing whether the amplitude of the fitted curve was significantly (p < 0.05) different from zero: Following Cornelissen^40^, a linear expression of a single phase Cosinor model of a value measured at time “t” is given by:

$$Y(t)=M+\beta x+\gamma z+e(t)$$

where

$$\beta =Acos\phi ;\gamma =-\,Asin\phi ;x=\,\cos (\frac{2\pi t}{T});z=\,\sin (\frac{2\pi t}{T}),$$

$$A\,is\,the\,amplitude\,of\,the\,curve\,and\,\phi \,the\,acrophase.$$

It therefore follows that if either coefficient, $\beta \,or\,\gamma$ are significantly different from 0, the amplitude, A, is significantly different from 0, hence establishing a statistically significant single phase cosine pattern to the data.

$${\boldsymbol{A}}=\sqrt{{{\boldsymbol{\beta }}}^{2}+{{\boldsymbol{\gamma }}}^{2}}$$

$\phi =\arctan (-{\boldsymbol{\gamma }}/{\boldsymbol{\beta }})$Text in bold denotes an estimate of the parameter. The resultant parameter estimates and accompanying bootstrapped 95% confidence intervals are given in Supplemental Table 4. Bootstrapping was carried out in R.

In fitting the linear model above, we included a random effect for subject in order to account for the repeated measurements taken from each subject. This was accomplished using SAS Procedure “Mixed”. In addition, we tested whether cortisol influenced circadian rhythmicity by fitting the linear model both with and without a term for cortisol. The results from this analysis are presented in Supplemental Table 3 are coded as “yes” (statistically significant, p < 0.05) and “no” (not statistically significant).

### Potential limitations

Possible limitations of our methodology include: (1) our model of circadian rhythmicity is perhaps too simple. For all components of the normal immune system, we fit a cosine curve having a 24-hour period. There might actually be shorter periods in some components. Yet, we felt that a thorough investigation of this question might, however, not be as informative as the simple approach taken in this study. More sophisticated models could be examined in the future as a separate effort to develop methodologies; (2) we used Pearson correlation to investigate relationships among immune components. Although it might be preferable to use a distribution-free statistic, such as the Spearman correlation, we elected to use Pearson given the fact we had n = 54 data points for estimating each correlation (after adjusting for with-subject correlation) which we feel is sufficient to make the distinction between these two methods inconsequential. We also point out that we did not use the p-values when adjusting for false-discovery, but that we used the actual correlation estimates instead. Hence our estimates and statistical interpretations of correlations are based on an unbiased estimator from a moderately-large sample size; (3) our “detrended” data is generated as the residuals after fitting a cosine model to the data. Hence, the precision of this data set, and possible bias, is a function of model fit. Factors such as model complexity and sample size need to be investigated in order to determine better methods if possible for extracting circadian variation; (4) we decided to focus on percent rather than count data. Since percent data can be affected by the quantity of other data, we recognize this as a limitation arising from our desire to use a conservative rule when identifying “significant” parameters.

## Supplementary information

Supplementary Information
