# Supplementary material for: Synchronization of the Normal Human Peripheral Immune System: A Comprehensive Circadian Systems Immunology Analysis
Source: Sci Rep. 2020 Jan 20;10:672. doi: 10.1038/s41598-019-56951-5 (PMC6971086; doi:10.1038/s41598-019-56951-5)
Supplement: Supplementary file 1 — Supplementary Information [file 41598_2019_56951_MOESM1_ESM.docx]

**-SUPPLEMENTARY INFORMATION-**

**Synchronization of the Normal Human Peripheral Immune System: A Comprehensive Circadian Systems Immunology Analysis**

Running title: Circadian Systems Immunology

Craig A. Beam, PhD^1^*, Clive Wasserfall, PhD^2^, Alyssa Woodwyk, MS^1^, McKenzie Akers, MD^1^, Heather Rauch^1^, Thomas Blok, MD^3^, Patrice Mason, RN^3^, Duncan Vos, MS^1^, Daniel Perry, PhD^2^, Todd Brusko, PhD^2^, Mark Peakman, PhD^4^, Mark Atkinson, PhD^2^

^1^Department of Biomedical Sciences, Western Michigan University Homer W. Stryker M.D. School of Medicine

^2^Department of Pathology, Immunology, and Laboratory Medicine, University of Florida Diabetes Institute, Gainesville, FL

^3^Center for Clinical Research, Western Michigan University Homer W. Stryker M.D. School of Medicine

^4^Department of Immunobiology, Faculty of Life Sciences & Medicine, King’s College London, 2nd Floor, Borough Wing, Guy's Hospital, London, SE1 9RT, UK.

**Supplementary Table 1 Subject Demographics**

| **Subject Number** | **Age** | **Gender** | **Height**  **(cm)** | **Weight**  **(kg)** | **Body Mass Index**  **(kg/m^2^)** | **Temperature at Enrollment (C)** |
| --- | --- | --- | --- | --- | --- | --- |
| **1** | **38** | **F** | **158.8** | **76.8** | **30.46** | **36.7** |
| **2** | **31** | **M** | **177** | **81** | **25.85** | **36.7** |
| **3** | **38** | **M** | **179.4** | **111.3** | **34.58** | **36.3** |
| **4** | **24** | **M** | **176.5** | **73.8** | **23.69** | **36.7** |
| **5** | **38** | **F** | **158.7** | **62** | **24.62** | **36.3** |
| **6** | **22** | **F** | **170** | **92.4** | **31.97** | **37** |
| **7** | **31** | **M** | **177.2** | **83.9** | **26.72** | **36.5** |
| **8** | **35** | **F** | **149.2** | **67.4** | **30.28** | **36.6** |
| **9** | **31** | **M** | **177.85** | **95.1** | **30.07** | **36.6** |
| **10** | **32** | **M** | **188.2** | **103** | **29.08** | **36.8** |

**Supplementary Table 2.** **Inventory of Peripheral Correlative and Phase Relationships.** Correlations of immune-related circulatory cell populations and cytokines after ordering on the sequence of their peak appearance during a 24-hour observation period. Correlations between pairs of immune variables whose peaks occur one after the other ("Phase Adjacency") are highlighted in bold text and border and appear above/below the diagonal. Significant (FDR-adjusted) correlations are italicized. Time of day of peak occurrence during the day of the study is indicated in the left-most column.

**Supplementary Table 3.a** **Statistical significance of cosinor models.** As described in Online Methods, the cosinor model (see **Supplemental Figure 1**) is fit with two parameters designated in the table as "β" and "γ". Statistical significance of each of these parameters at p<0.05 are denoted in the table with "Yes". "No" means the test was not significant. Results are presented without and with an adjustment for serum cortisol level in order to identify variables whose circadian rhythmicity was influenced by cortisol level, a hormone of the neuroendocrine system.

| **Immune factor (%)** | **β**  **without Cortisol adjustment** | **γ**  **without Cortisol**  **adjustment** | **β**  **with Cortisol**  **adjustment** | **γ**  **with Cortisol**  **adjustment** |
| --- | --- | --- | --- | --- |
| Basophil | No | No | No | No |
| B cell | No | No | No | No |
| CD4^-^CD8^-^ | Yes | No | No | No |
| CD4^+^CD8^+^ | No | Yes | No | Yes |
| CD4^+^ | Yes | Yes | No | Yes |
| CD56^bright^ NK | No | Yes | No | Yes |
| CD56^dim^ NK | No | Yes | No | Yes |
| CD8^+^ | No | No | No | No |
| Classical monocytes | No | Yes | No | Yes |
| DC | No | Yes | No | No |
| Eosinophil | No | No | No | Yes |
| Granulocyte leukocytes | Yes | No | Yes | No |
| HCT | No | Yes | No | No |
| HGB | No | Yes | No | No |
| Lymphocytes | Yes | No | Yes | No |
| Lymph monocytes | Yes | No | Yes | No |
| Monocytes | Yes | No | Yes | No |
| Naïve CD4^+^ | Yes | No | No | No |
| Naïve CD8^+^ | No | Yes | No | Yes |
| Neutrophil | Yes | No | Yes | No |
| NK | No | Yes | No | Yes |
| NKT | No | No | No | No |
| Plasma IFN-γ | No | No | No | No |
| Plasma IL-12 | No | Yes | No | Yes |
| Plasma IL-2 | No | No | No | Yes |
| Plasma IL-4 | Yes | Yes | Yes | Yes |
| Plasma IL-6 | No | No | No | No |
| Plasma sIL-2Ra | No | Yes | Yes | Yes |
| Plasma sIL-4R | No | Yes | No | Yes |
| Plasma sIL-6R | No | Yes | No | Yes |
| Platelets | No | No | Yes | No |
| Serum IFN-γ | No | No | No | No |
| Serum IL-12 | No | No | No | No |
| Serum IL-2 | No | No | Yes | No |
| Serum IL-4 | No | No | No | No |
| Serum IL-6 | No | No | No | No |
| Serum sIL-2ra | No | Yes | No | Yes |
| Serum sIL-4r | Yes | No | Yes | No |
| Serum sIL-6r | No | No | No | No |
| Tcm CD4^+^ | Yes | No | Yes | No |
| Tcm CD8^+^ | Yes | No | Yes | No |
| TEM CD4^+^ | No | Yes | No | No |
| TEM CD8^+^ | Yes | Yes | Yes | Yes |
| TEMRA CD4^+^ | Yes | Yes | Yes | No |
| TEMRA CD8^+^ | Yes | Yes | No | Yes |
| Treg | No | Yes | No | Yes |
| Unidentified lymphocytes | No | Yes | No | Yes |
| WBC | Yes | No | Yes | No |
| Nonclassical monocytes | No | Yes | No | Yes |

**Supplementary Table 3.b P-values related to Supplementary Table 2.a.** P-values are from the analysis conducted without inclusion of cortisol.

|  | **P-value** | |
| --- | --- | --- |
| **Immune Factor** | **β** | **γ** |
| basophils | 0.191497 | 0.949473 |
| B cell | 0.086942 | 0.394939 |
| CD4+ | 0.000499 | 0.48342 |
| CD56bright NK | 0.001913 | 0.002807 |
| CD56dim NK | 0.001761 | 0.002682 |
| CD8 | 0.742401 | 0.020137 |
| Classical Monocytes | 0.001144 | 0.027084 |
| cortisol | 0.000356 | 2.11E-05 |
| DC | 0.048918 | 0.272016 |
| eosinophils | 0.176789 | 0.21451 |
| granulocytes | 0.000185 | 0.059862 |
| Lymphocytes | 0.02228 | 0.474086 |
| Lymph Mono | 0.000199 | 0.060727 |
| Monocyte | 0.009477 | 0.939814 |
| Naïve CD4Tconv | 0.844605 | 0.008872 |
| Naïve CD8Tcells | 0.002212 | 0.007273 |
| Neutrophils | 0.012277 | 0.305278 |
| NK | 0.002579 | 0.542329 |
| NKT | 0.415883 | 0.355979 |
| Plasma_IFNγ | 0.240262 | 0.395773 |
| Plasma IL12 | 0.007934 | 0.17521 |
| Plasma IL2 | 0.070024 | 0.436031 |
| Plasma IL4 | 1.1E-05 | 0.157224 |
| Plasma IL6 | 0.131231 | 0.689358 |
| Plasma sIL2Ra | 3.75E-08 | 0.000674 |
| Plasma sIL4R | 0.04522 | 0.284864 |
| Plasma sIL6R | 0.00067 | 0.010754 |
| Serum_IFNγ | 0.278814 | 0.26048 |
| Serum IL12 | 0.913856 | 0.100686 |
| Serum IL2 | 0.315908 | 0.37187 |
| Serum IL4 | 0.181764 | 0.172724 |
| Serum IL6 | 0.544165 | 0.709614 |
| Serum sIL2Ra | 0.050705 | 0.020345 |
| Serum sIL4R | 0.031441 | 0.12899 |
| Tcm CD4Tconv | 0.031892 | 0.000202 |
| Tcm CD8Tcells | 3.56E-07 | 1.16E-05 |
| Tem CD4Tconv | 0.00387 | 0.458638 |
| Tem CD8Tcells | 5.01E-05 | 0.378637 |
| Temra CD4Tconv | 0.001197 | 0.883582 |
| Temra CD8Tcells | 0.000117 | 0.752078 |
| Treg | 0.00123 | 0.043817 |

**Supplementary Table 4 Cosinor Model Parameter Estimates.** Parameter estimates from the linear model described in Online Methods.

| **Analyte** | **A** | **95% CI** | **Φ** | **95% CI** | **M** | **95% CI** |
| --- | --- | --- | --- | --- | --- | --- |
| **Bcell** | **0.3392** | **0.1013,0.7074** | **-5.8263** | **-6.236,-0.054** | **1.34E-16** | **-0.3623,0.3579** |
| **CD4** | **0.6046** | **0.3278,0.9346** | **-0.1876** | **-6.263,-0.023** | **6.92E-16** | **-0.3767,0.3550** |
| **CD4+CD8+** | **0.6685** | **0.4049,0.9773** | **-0.5594** | **-1.087,-0.113** | **6.96E-17** | **-0.2672,0.2811** |
| **CD4m-CD8-** | **0.5247** | **0.2499,0.8778** | **-2.9682** | **-3.663,-2.292** | **3.21E-16** | **-0.2954,0.2966** |
| **CD56bright NK** | **0.6922** | **0.4019,1.0090** | **-0.7647** | **-1.248,-0.338** | **1.98E-17** | **-0.2401,0.2405** |
| **CD56dim NK** | **0.6956** | **0.4079,1.0108** | **-3.9045** | **-4.397,-3.480** | **-3.98E-15** | **-0.2330,0.2366** |
| **CD8** | **0.4146** | **0.1569,0.7741** | **-1.4339** | **-2.417,-0.472** | **-7.10E-16** | **-0.3196,0.3053** |
| **Classical Monocytes** | **0.6464** | **0.3831,0.9535** | **-0.5836** | **-1.236,-0.134** | **1.79E-15** | **-0.3071,0.3133** |
| **DC** | **0.3962** | **0.1547,0.7511** | **-3.6447** | **-4.619,-2.650** | **2.19E-17** | **-0.2964,0.2956** |
| **Eosinophils** | **0.3252** | **0.1138,0.6782** | **-0.7425** | **-6.200,-0.082** | **-1.35E-16** | **-0.3714,0.3518** |
| **Granulocytes** | **0.6848** | **0.4257,0.9806** | **-2.6963** | **-3.207,-2.235** | **1.61E-16** | **-0.3723,0.3676** |
| **Naïve CD4** | **0.4608** | **0.1608,0.8321** | **-1.6431** | **-2.404,-0.780** | **-9.44E-16** | **-0.4554,0.4325** |
| **Naïve CD8** | **0.6622** | **0.4315,0.9481** | **-0.7146** | **-1.252,-0.205** | **-5.92E-16** | **-0.5116,0.5569** |
| **NK** | **0.5318** | **0.2682,0.8704** | **-3.3327** | **-4.018,-2.645** | **4.30E-17** | **-0.3434,0.3709** |
| **NKT** | **0.2211** | **0.0619,0.6234** | **-3.9904** | **-5.567,-1.485** | **-5.47E-17** | **-0.3915,0.3839** |
| **Plasma IFNg** | **0.2594** | **0.0646,0.6664** | **-3.7661** | **-5.391,-1.232** | **-2.84E-16** | **-0.3038,0.3012** |
| **Plasma IL 12** | **0.5122** | **0.2331,0.8716** | **-3.6032** | **-4.382,-2.949** | **-6.32E-16** | **-0.2538,0.2519** |
| **Plasma IL 2** | **0.3498** | **0.1023,0.7276** | **-3.5428** | **-4.790,-2.246** | **-1.16E-16** | **-0.2985,0.3041** |
| **Plasma IL 4** | **0.7486** | **0.5345,1.0202** | **-3.4280** | **-3.907,-3.025** | **-7.26E-16** | **-0.2138,0.2182** |
| **Plasma IL 6** | **0.2798** | **0.0729,0.6901** | **-2.8853** | **-4.870,-1.386** | **1.02E-15** | **-0.3149,0.3058** |
| **Tcm CD4** | **0.6961** | **0.4233,0.9959** | **-5.2159** | **-5.671,-4.761** | **-2.38E-16** | **-0.3359,0.3452** |
| **Tcm CD8** | **0.9410** | **0.7167,1.1822** | **-5.5895** | **-5.863,-5.310** | **-3.13E-16** | **-0.3316,0.3279** |
| **Tem CD4T** | **0.5165** | **0.2585,0.8607** | **-3.3835** | **-4.139,-2.694** | **1.20E-16** | **-0.4473,0.4089** |
| **Tem CD8** | **0.6902** | **0.4565,0.9967** | **-3.3394** | **-3.833,-2.840** | **1.14E-16** | **-0.4202,0.4094** |
| **Temra CD4** | **0.5596** | **0.2426,0.9272** | **-3.1844** | **-3.774,-2.586** | **2.33E-16** | **-0.3163,0.3305** |
| **Temra CD8** | **0.6528** | **0.3582,0.9834** | **-3.2176** | **-3.742,-2.751** | **-7.36E-17** | **-0.4929,0.5008** |
| **Treg** | **0.6307** | **0.3442,0.9765** | **-0.5433** | **-1.227,-0.127** | **-3.08E-16** | **-0.4420,0.4238** |

**Symbol Key:** A=Amplitude; Φ=Acrophase; M=MESOR.

Acrophase estimates are expressed as they appear in the linear COSINOR model and are expressed as radians. The conversion formula to 24 hour clock time is: $clock time \left( hrs \right)=\left| acrophase \right|*({24}/{2\pi})$.

**Supplementary Table 5. Key to Figure 4**

| **Key** | **Factor** | **Key** | **Factor** |
| --- | --- | --- | --- |
| 1 | granulocytes | 29 | basophils |
| 2 | neutrophils | 30 | CD4 T |
| 3 | CD4-CD8- | 31 | CD4+CD8+ |
| 4 | monocytes | 32 | classical monocytes |
| 5 | Temra CD4 | 33 | Regulatory T |
| 6 | Temra CD8 | 34 | naïve CD8 |
| 7 | NK | 35 | eosinophils |
| 8 | Tem CD8 | 36 | NK CD56bright |
| 9 | Tem CD4 | 37 | RDW |
| 10 | DC | 38 | CD8 T |
| 11 | unidentified lymphocytes | 39 | Naïve CD4 |
| 12 | non-classical monocytes | 40 | serum IFNγ |
| 13 | MCV | 41 | serum IL-12 |
| 14 | NKT | 42 | serum IL-2 |
| 15 | HCT | 43 | serum IL-4 |
| 16 | NK CD56dim | 44 | serum IL-6 |
| 17 | RBC | 45 | serum sIL-2Ra |
| 18 | HGB | 46 | serum sIL-4R |
| 19 | MPV | 47 | serum-sIL-6R |
| 20 | platelets | 48 | plasma IFNγ |
| 21 | Tcm CD4 | 49 | plasma IL-12 |
| 22 | Tcm CD8 | 50 | plasma IL-2 |
| 23 | B-cell | 51 | plasma IL-4 |
| 24 | monocytic lymphocytes | 52 | plasma IL-6 |
| 25 | MCH | 53 | plasma sIL-2Ra |
| 26 | lymphocytes | 54 | plasma sIL-4R |
| 27 | WBC | 55 | plasma-sIL-6R |
| 28 | monocytes | 56 | cortisol |

**
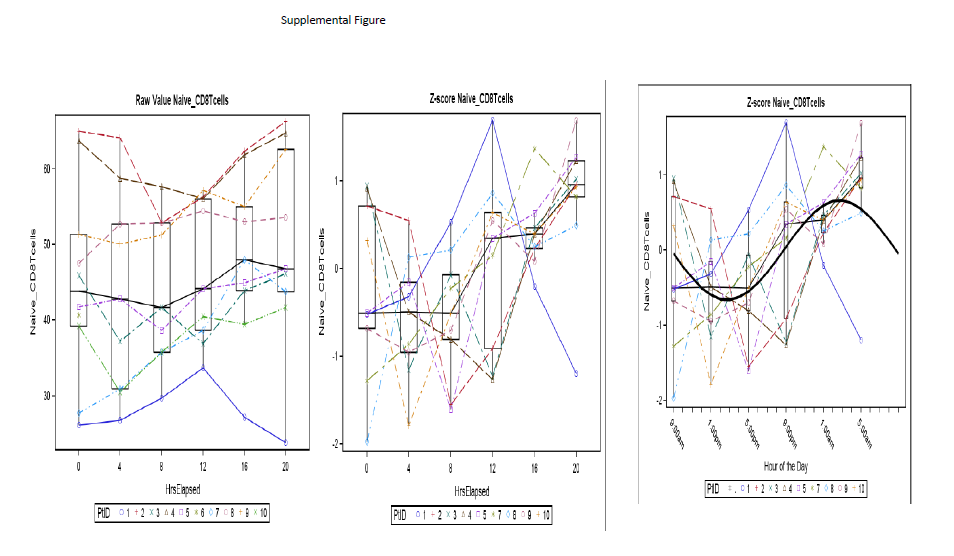
Supplementary Figure 1. Examples of data and fitted cosine.**

**Supplementary Figure 2 Flow cytometry gating.**

**Supplementary Figure 3 Circadian Patterns of Cell Population Counts.** Box plots of white blood cell counts (panel A) and lymphocyte counts (B) variation for each of the 9 study subjects with superimposed cosine curve representing average circadian rhythmicity are presented. Within each box plot the median is presented by a horizontal bar and medians are connected by line segments. In addition, the data from each subject are connected with dashed lines.


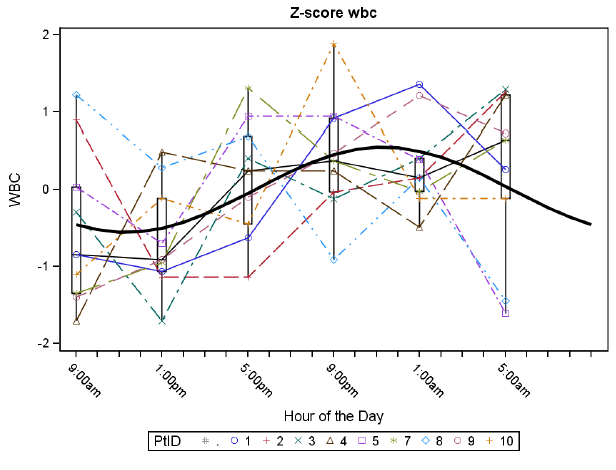

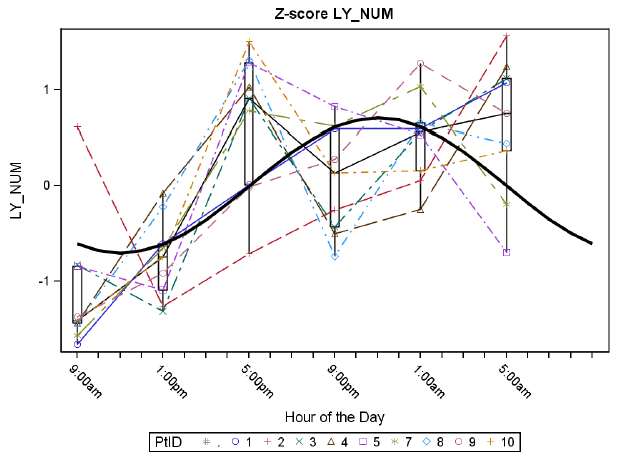


**B**

**A**
